# Dipolar colloids in apolar media: direct microscopy of two-dimensional suspensions

**DOI:** 10.1038/srep28578

**Published:** 2016-06-27

**Authors:** Erez Janai, Avner P. Cohen, Alexander V. Butenko, Andrew B. Schofield, Moty Schultz, Eli Sloutskin

**Affiliations:** 1 Physics Department and Institute for Nanotechnology & Advanced Materials, Bar-Ilan University, Ramat-Gan 5290002, Israel; 2 School of Physics and Astronomy, University of Edinburgh, Edinburgh EH9 3FD, United Kingdom

## Abstract

Spherical colloids, in an absence of external fields, are commonly assumed to interact solely through rotationally-invariant potentials, *u*(*r*). While the presence of permanent dipoles in aqueous suspensions has been previously suggested by some experiments, the rotational degrees of freedom of spherical colloids are typically neglected. We prove, by direct experiments, the presence of permanent dipoles in commonly used spherical poly(methyl methacrylate) (PMMA) colloids, suspended in an apolar organic medium. We study, by a combination of direct confocal microscopy, computer simulations, and theory, the structure and other thermodynamical properties of organic suspensions of colloidal spheres, confined to a two-dimensional (2D) monolayer. Our studies reveal the effects of the dipolar interactions on the structure and the osmotic pressure of these fluids. These observations have far-reaching consequences for the fundamental colloidal science, opening new directions in self-assembly of complex colloidal clusters.

Suspensions of colloidal spheres in low polarity and apolar solvents have been intensively studied in the last decade as a model system^1^,^2^,^3^,^4^,^5^,^6^,^7^, mimicking the phase behavior of atoms and molecules. Apolar colloidal and nanoparticle suspensions have important applications in electrophoretic displays, in printing and copying technologies, as also in electrorheology^8^. Yet, while the colloidal interaction potentials in aqueous media are reasonably-well understood, even the most fundamental concepts of colloidal interactions in organic media are still being developed^8^,^9^,^10^. Moreover, while some indications exist for the presence of permanent electric^11^,^12^,^13^,^14^,^15^,^16^ and magnetic^17^ dipoles on spherical colloids and nanoparticles in *aqueous* media, the possible presence of permanent electric dipoles in *organic* suspensions of colloidal spheres has never been tested.

We demonstrate, by direct experiments, that poly(methyl methacrylate) (PMMA) colloidal spheres^18^, suspended in an apolar solvent, bear permanent electric dipoles. In particular, we demonstrate that the Brownian rotations of such spheres in an external DC electic field are limited by the dipole-field interaction. Furthermore, we employ direct optical microscopy and a corrected particle location algorithm^19^ to study the structure of a 2D colloidal fluid of such spheres, in a wide range of particle concentrations. We demonstrate that the structure and the thermodynamics of these fluids cannot be matched by computer simulations, if the interactions are assumed to be either hard- or soft-repulsive; simple point dipole simulations are in a qualitative agreement with the experimental trends. Electric dipoles give rise to short range attractions, increasing the abundance of particle pairs; with the dipoles in the present work being relatively weak, only transient particle pairs are observed, constantly forming and dissociating. It is yet unclear, whether the highly-controversial like-charged particle attractions in two-dimensional *aqueous* suspensions^20^,^21^,^22^ are driven by a similar mechanism^15^. Finally, the presence of the dipolar interactions significantly increases the range of the bond-orientational order in these fluids, suggesting that the dipoles may possibly be promoting the transition to an hexatic phase^23^,^24^,^25^. Our observations demonstrate that the dipolar interactions introduce a non-negligible coupling between rotations and translations of the spherical colloids in organic suspensions; this fact has long-reaching consequences for both the fundamental physics of such colloids and for their various applications.

Colloidal spheres suspended in a solvent explore their phase space by random Brownian motion. As a result, fluid colloidal suspensions tend to minimize their free energy, akin to conventional fluids of atoms and molecules^1^,^2^,^7^. However, being much larger than the molecules, colloids are readily visible by conventional optical microscopy, allowing their positions to be detected in real motion, which is practically impossible with fluids of atoms and molecules^3^,^7^. The availability of such highly-detailed experimental information, as also the tunability of pair-potentials in colloidal suspensions^2^, make colloids a unique model system, providing a deeper insight into the fundamental physics of collective phenomena in fluids^3^,^4^,^5^,^6^,^7^,^26^. Moreover, the studies of colloids are by no means restricted to three-dimensional systems; colloids can readily be confined, through introduction of either solid^24^,^27^ or liquid boundaries^28^,^29^, to a 2D space, thus forming a very simple physical model of a Langmuir film (LF), where molecular degrees of freedom are similarly constrained to two spatial dimensions^30^. The phase behavior of 2D systems is typically very different from that of the bulk matter. In particular, recent computer simulations^23^,^25^ of hard disks (HDs) suggest a phase transition from a fluid to an hexatic phase to occur when the area fraction of the disks reaches *η* ≈ 0.704; the corresponding number density is *ρ* = 0.896 *σ*^−2^, where *σ* is the particle diameter. This transition, where the spatial correlations between the nearest-neighbor (NN) bond orientations become quasi-long-ranged, was demonstrated to be of the first-order, followed by a second-order transition at *η* ≈ 0.72 to a two-dimensional solid. These new theoretical results, following decades of fierce debates over the nature of phase transitions in HDs^23^, pose a whole range of new questions. How sensitive is this phase diagram to slight deviations from the ideal HD interactions? What metastable states may occur? Moreover, the phase diagram of HDs was demonstrated to be notoriously sensitive to finite size effects^23^, significantly challenging further progress by computer simulations. Colloidal experiments, where sample size is essentially unlimited, provide promising pathways for exploration of 2D fluids and solids of hard, or nearly-hard particles. However, only in a few experimental works were the structures of monodisperse colloidal 2D fluids systematically studied, over a wide range of *η* values^24^,^31^,^32^. Most of these studies were done more than a decade ago. The image analysis in some of these works has being carried out on computer architectures that have since been superseded. At least in some cases, the accuracy of particle location algorithms was traded for the speed of computation, which could lead to inaccurate radial distribution functions and artificial long range orientational correlations^19^. Finally, the experimentally-determined fluid structures have not been compared to theoretical predictions, so that a full quantitative understanding of the observed structures was not established. In order to fill this gap, in present work we detect particle positions by a corrected particle location algorithm^19^ and directly compare the experimental fluid structures with computer simulations.

## Results and Discussion

**Figure 1:**
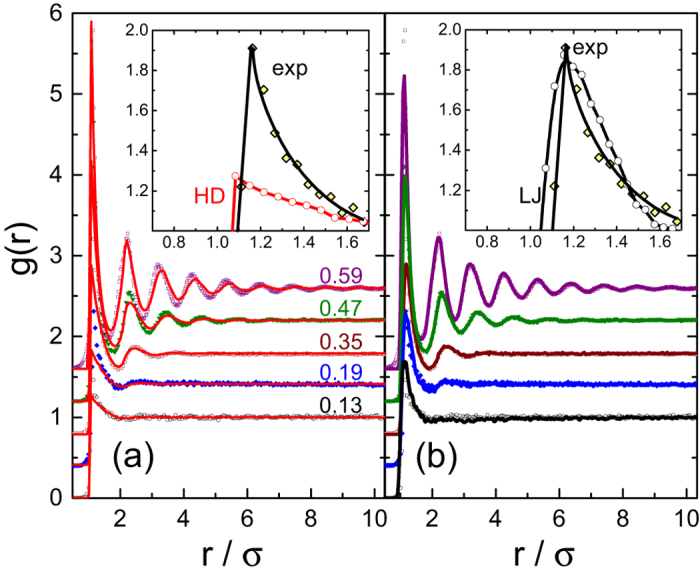
Experimental radial distribution functions (scatter) for a range of particle area fractions *η*; see the *η* values marked on the graph. The plots are shifted vertically for clarity of representation. The solid curves represent the simulated *g*(*r*), obtained (**a**) by event-driven Brownian dynamics of HDs (**b**) by molecular dynamics of LJ particles. Note the much better quality of the fits in (**b**). The insets zoom onto the principal peak of the *g*(*r*) for *η* = 0.19, to emphasize the mismatch between our experimental data (exp) and hard disks (HD) simulations. The mismatch is much smaller for the LJ potentials [section (**b**)], suggesting that the experimental pair potentials include an attractive component, shown in the following to be resulted by the electric dipoles.

**Figure 2:**
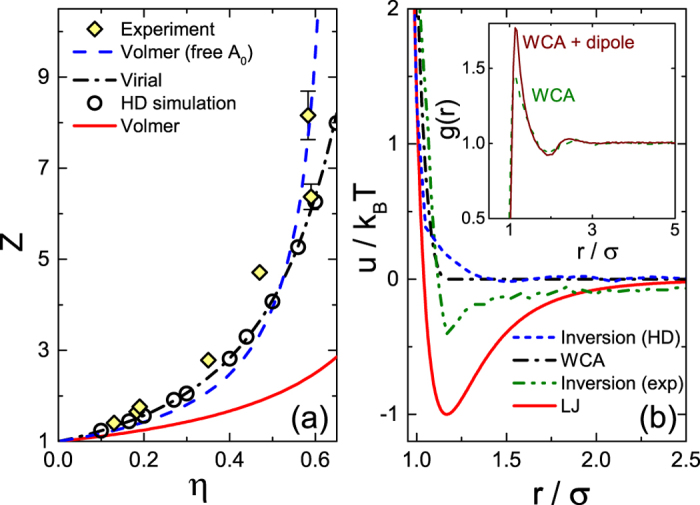
(**a**) The experimental compressibility factor *Z* (rhombi), obtained under the assumption of hard rotationally-isotropic interaction potentials, is completely missed by the Volmer isotherm (solid red line). The agreement is poor for even an unphysical *A*_0_ value (blue dashes). A much better match, with no free parameters, is achieved for virial expansions (dash-dotted curve) and HDs simulations (circles). Note, since the deduction of *Z* from our experiments assumes hard interactions, we do not compare these *Z* to WCA or LJ fluid simulations, with the later^48^ strongly deviating from the rhombi. As the experimental error bars on *η* are of about the symbol size, the *η* of the two rightmost rhombi coincide within error. (**b**) The *u*(*r*) used in WCA and LJ simulations are displayed on top of the *u*(*r*) obtained by inversion of the experimental *g*(*r*), for *η* = 0.19; note the presence of an attraction well in the last (olive dash-dot-dot curve). Here we use 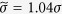 for WCA and LJ potentials, as in the corresponding *g*(*r*) fits, where *σ* is the experimental particle size measured by light scattering, as in previous work^4^. For comparison, no attractions are observed when the inversion algorithm is applied to EDBD-simulated *g*(*r*) of ideal HDs, at *η* = 0.44 (short blue dashes). **Inset:** The *g*(*r*) of a simulated zero-dipole purely-repulsive WCA fluid (dashes; *ρ* = 0.31) is compared to that with point dipoles (solid curve; the dipole moment is |*p*| = 1.5 in the LJ units^37^ and *ρ* = 0.2). Note the much higher first peak in the dipolar fluid, indicative of an enhanced pair formation. The higher-order peaks have roughly the same amplitude. A similar increase in the first peak of the *g*(*r*), with unchanged higher-order peaks, was observed for the experimental fluids, when compared to the purely repulsive (HD or WCA) simulations. Thus, while a more advanced model, beyond the point dipole approximation, is necessary to fully reproduce the experimental *g*(*r*), our simple simulations are successfully imitating the qualitative trends observed in the experiment.

To quantitatively characterize the structure of the 2D fluids of PMMA colloids in an apolar solvent, we employ the radial distribution function *g*(*r*), obtained from the experimentally-detected positions of the individual particles (left panel of Video 1 in the Supplementary Material). For a given particle area fraction *η, g*(*r*) is the probability to find two particles at a separation *r*, normalized by the same probability in an ideal gas, where particles interpenetrate and correlations are completely missing. In fluids, where no long range correlations are present, *g*(*r* → ∞) = 1, as demonstrated by the experimental *g*(*r*) in Fig. 1. The oscillations of *g*(*r*), representing the coordination shell structure of the fluid^4^, characterize the thermodynamical properties of the colloidal fluid. In particular, for hard rounded particles, the osmotic pressure is given by^23^ *βP* = *ρ*[1 + 2*ηg*(*r* → *σ*^+^)], where *β* ≡ (*k*_*B*_*T*)^−1^. To work with non-dimensional quantities, we replace *P* by the compressibility factor *Z* = *βPρ*^−1^, which relates the pressure of our experimental colloidal fluid to that of an ideal gas. The experimental *Z* values, obtained from *g*(*r*) as above (under the assumption of hard interactions), increase with *η*, as shown in Fig. 2(a) (rhombi). Particle correlations are negligible at low densities, as in an ideal gas, so that *Z*(*η* → 0) = 1. Pressure isotherms of colloids have been previously obtained in three dimensions (3D), by either confocal microscopy^33^ or direct compression by a piston^34^. Also, the pressure of 2D films of colloids floating at liquid interfaces, where complex capillary-wave-mediated interactions take place, has been measured by a direct compression in a Langmuir trough^29^. We are not aware of *Z*(*η*) measurements carried out for 2D colloidal fluids on a solid substrate; therefore, we compare our results to the theoretical Volmer isotherm: *βP* = (*A* − *A*_0_)^−1^, where *A* = *ρ*^−1^ is the area per one colloidal particle and *A*_0_ ≈ *πσ*^2^/4 is the excluded area. With no free parameters involved in this comparison, the Volmer isotherm completely misses the experimental data [solid red line in Fig. 2(a)]; indeed, this isotherm predicts a finite *P* for densities exceeding the close packing density of disks on a plane. A much better match to the experimental data results if *A*_0_ is tuned as a free parameter [blue dashes in Fig. 2(a)]; however, the fitted *A*_0_ ≈ 6.8 *μm*^2^ exceeds the experimental particle cross-section area by a factor of 1.5, which is far too large to be accounted for by the presence of voids in either the hexagonal (*η* ≈ 0.907) or the random (*η* ≈ 0.82) close packings of disks^35^. Moreover, this adapted Volmer isotherm still does not match the experiment very well, exhibiting negative and positive deviations at intermediate and high *η*, respectively. Similar deviations have been observed for molecular Langmuir films^30^ and attributed to possible inaccuracies of the phenomenological Volmer isotherm.

To test the accuracy of the Volmer isotherm, we compare it to a simulated *Z*(*η*) of a system of ideal hard disks. For that purpose, we obtain the *g*(*r*) of hard disks (HDs) by event-driven Brownian dynamics (EDBD) simulations; *Z*(*η*) are then obtained from the height of the principal peak of *g*(*r*), as above. The resulting *Z*(*η*) [open circles in Fig. 2(a)] are still missed completely by the Volmer isotherm, for either a fixed or a free excluded area *A*_0_. However, our simulations are in a perfect agreement with the virial expansions (dash-dotted curve), obtained by a direct numerical calculation^36^. This fact strongly supports the validity of our simulations and confirms that the phenomenological Volmer isotherm is quite inaccurate even for this simplest system of simulated HDs, where only excluded volume interactions are present. The agreement of either the virial expansion calculations or hard disk simulations with the experimental *Z*(*η*), with no adjustable parameters, is much better than for the Volmer isotherm [Fig. 2(a)]; however, the deviations with the experiments are still non-negligible, suggesting that an additional insight may be gained by a direct comparison between the full experimental *g*(*R*) and computer simulations.

While the isotherms of HDs in Fig. 2(a) exhibit only slight deviations from the experiment, the agreement between the corresponding experimental and theoretical *g*(*r*) functions is worse. For the HD simulations to match the experimental *g*(*r*), we had to increase *σ* by a factor of 1.05, well beyond the experimental error bar on this value; this factor was kept the same for all *η*. The rescaled *σ* implies that the effective area fraction is higher than the experimental one; thus, the *η* values were rescaled accordingly by a factor of 1.05^2^ = 1.10. This rescaling of *σ* and *η* could potentially be justified by a possible presence of Coulomb repulsions between the colloids^4^,^26^. However, even with this rescaling employed, the match between the experimental and the theoretical *g*(*r*) is far from being perfect. Particularly strong mismatches are observed for the principal peak; see the zoom in the inset to Fig. 1(a). These observations, which cannot be masked by a simple rescaling of *σ*, indicate that the interparticle potentials are qualitatively different from these of the HDs.

**Figure 3:**
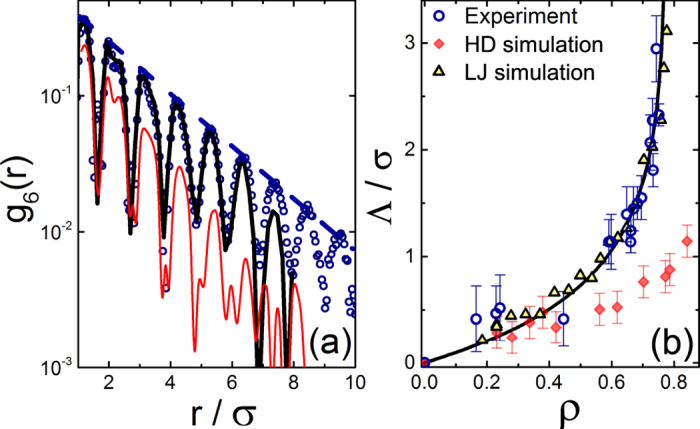
(**a**) Experimental (circles) bond-orientational correlation functions *g*_6_(*r*), at *η* = 0.59, nicely overlap with the LJ-simulated *g*_6_(*r*) (black curve); where *ρ* and *σ* are the same as for the positional *g*(*r*) functions in Fig. 1(b), with no additional adjustable parameters involved. A significant disagreement with the experiments occurs for the HDs simulations (red curve). The amplitudes of *g*_6_(*r*) exhibit an exponential decay (royal blue dashes), yielding the bond-orientational correlations Λ, shown in (**b**); the particle densities *ρ* are in the *σ*^−2^ units. Note the divergence of Λ at *ρ* ≈ 0.78, much lower than the liquid-hexatic (or hexatic-solid) transition of the HDs. Accordingly, the Λ values obtained in the HDs simulations (red rhombi) significantly underestimate the experimental ones (circles). The LJ-simulated Λ (triangles) perfectly match with the experiment. The error bars corresponding to the triangles are of the same size as for the circles; for the clarity of representation, we do not show these error bars.

An even more stringent test of the HDs model is provided by the orientational correlations of the bonds between the NNs. To quantify these correlations, we employ the two-dimensional local bond-orientational order parameters^1^:


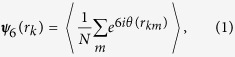


where the index *m* counts the *k*th particle’s NNs, *θ*(*r*_*km*_) is the angle between the bond connecting particles *k* and *m* and an arbitrary fixed reference axis, and *N* is the number of *k* − *m* bonds. Here *r*_*k*_ denotes the location of the *k*th particle. With a complex *ψ*_6_(*r*_*k*_) value attributed to each particle in the system, we obtain the spatial correlations of bond orientations as:


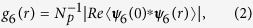


where *N*_*p*_ is the number of particle pairs separated by a distance *r* and the averaging is carried out over the whole sample^1^. The experimental *g*_6_(*r*) are shown in Fig. 3(a) (open symbols), with the semi-log scale emphasizing the exponential decay of the amplitude. The simulated *g*_6_(*r*) of the HDs (red curve), rescaled as for the *g*(*r*), strongly deviates from the experimental data, exhibiting a much stronger decay with *r*. The amplitudes of *g*_6_(*r*) (blue dashes) yield the bond-orientational correlation lengths Λ. Note the dramatic increase of Λ with the particle concentration, shown in Fig. 3(b). Remarkably, the simulated Λ of the HDs (red rhombi) strongly underestimate the experimental values (open circles). The solid curve in Fig. 3(b) is a phenomenological fit to the experimental data: 
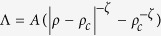
, where *A, ρ*_*c*_ and *ζ* are free fitting parameters. Interestingly, the fitted *ρ*_*c*_ = 0.78 ± 0.06 indicates a divergence of Λ occurring at a much lower density, compared to both the hexatic-crystal and the liquid-hexatic transitions of the ideal HDs^23^. This observation suggests that the interactions in our colloidal system are not hard and promote a stronger bond-orientational order. Moderate deviations from the ideally-hard repulsions, due to slight charging effects, could be taken into account by employing a softer WCA potential (see Methods)^4^,^37^, shown in Fig. 2(b). However, with the WCA potential employed, the deviations between the experiment and the simulation are not significantly reduced, indicating that the difference between the simulated and the experimental potentials goes beyond the common isotropic charging of the colloids. Also, we observe no electrophoretic motion of our particles under moderate external electric fields. Finally, the similarity of results obtained by either the molecular dynamics, employing WCA repulsions, or the event-driven Brownian dynamics of ideal HDs is an important indication for the discrepancy with the experiment being real, rather than a result of the simulation algorithm technicalities.

The rather poor match between the simulated purely-repulsive systems and the experimental data suggests that the particles’ distance of closest approach is set independently of the range and strength of particle correlations at larger separations. Such behavior may occur if the distance of closest approach is minimized by short range attractions, while the correlations at longer distances are mainly due to the particles’ mutual exclusion and repulsion. Zooming onto the principal peak of *g*(*r*) at low *η* further verifies this hypothesis: the HD simulations underestimate the experimental height of the peak [inset to Fig. 1(a)], indicating that the number of NNs per particle is increased beyond what is expected for a purely entropic system of hard disks. The number of NNs being increased beyond its random expectation value is a strong indication for short range attractions. Also, the experimental position of the principal peak is independent of *η*, which is not the case for soft repulsive potentials^4^. Finally, we invert the experimental *g*(*r*), using the classical Ornstein-Zernike formalism and the hypernetted chain (HNC) approximation to extract the pair potential by an iterative technique, described elsewhere^20^ (see Supplementary Information). While the resulting *u*(*r*) obtained for experimental samples at different *η* do not fully overlap, in all cases a potential well is clearly visible at short particle separations [see olive dash-dotted curve in Fig. 2(b)]; no similar potential well can be observed in *u*(*r*) obtained by inversion of simulated *g*(*r*) of ideal hard disks (short blue dashes)]. Thus, the combined evidence for presence of short range attractions in our 2D colloidal fluids is significant.

To obtain a better characterization of the short-range attractions, we carry out additional molecular dynamics (MD) simulations (right panel of Video 1 in the Supplementary Material), where full LJ potentials are used, instead of the repulsive WCA; here the LJ potential well accounts for the short-range attractions between the experimental colloids. The resulting fits are shown in Fig. 1(b). In these fits, 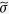 and the number density of the simulated particles *ρ*_*LJ*_ are tuned for a constant *T*, which is much less labor-intensive^4^; here 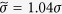 and *ρ*_*LJ*_/*ρ* = 1.0 ± 0.05 for all data. As expected, the presence of attractions allows the increased height of the principal peak of *g*(*r*) to be reproduced, as demonstrated in the inset. Indeed, the LJ simulations exhibit also an almost perfect agreement with the experimental *g*_6_(*r*), as shown in Fig. 3(a) (black curve). This agreement takes place at all particle concentrations, as evidenced by the perfect match of LJ-simulated Λ(*ρ*) [triangles in Fig. 3(b)] with the experiment. Finally, while the shape of the simulated LJ potential does not overlap with that of the *u*(*r*) obtained by an inversion of experimental *g*(*r*) at *η* = 0.19, the area of the curves in the range where *u*(*r*) < 1 differs by only <30%, providing a further support for the presence of short range attractions in these colloids.

**Figure 4:**
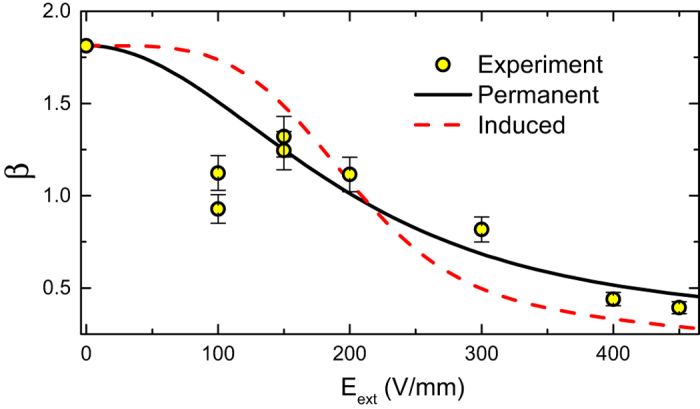
Brownian rotation of the colloids (at *η* → 0) is limited by the external electric field *E*_*ext*_. In particular, the standard deviation *β* of the projection of the dipole on the horizontal plane is reduced for high *E*_*ext*_, indicative of the presence of permanent dipoles. The theoretical fit, for a permanent dipole in a field, is shown with a solid curve. Note that the overall agreement of this model with the experimental data is better than for a more complex model, where an induced dipole couples to (possible) particle anisotropy (red dashes).

In general, the attractions between colloids^15^,^16^ or nanoparticles^38^ may be driven by several different mechanisms, among them the dipole-dipole interactions. In principle, dipole-dipole interactions may either be attractive or repulsive, depending on the coordinates of the interacting particles and the orientation of their dipoles. However, in fluids, where the particles are mobile, dipolar particles form clusters and chains, with the dominant dipole-dipole interactions always being attractive^15^,^16^,^38^. To directly confirm that the electric dipoles are indeed present in our system, we follow the Brownian rotation of an individual colloidal PMMA sphere in presence of an external electric field. For these experiments, we prepare a very dilute (*η* → 0) two-dimensional fluid as above, and sandwich the capillary between two electrodes, subject to a controllable DC electric potential difference, Δ*V*. With the edge effects neglected, the external electric field is *E*_*ext*_ = Δ*V*/*d*, where *d* = 2 mm is the width of the capillary; the actual field *E*_0_ at the particle location is lower due to the electrode polarization. To track the orientation of the particle, we first apply an electric field and bleach the fluorescent dye on one side of the particle, employing the full laser intensity of our confocal setup. With one side of the particle being darker than the other, its rotation in an external field is tracked by video-rate confocal microscopy (Video 2 in the Supplementary Material). Note, while a dipole is a vector in three dimensions, characterized by two angles: *θ* and *φ*, we only track the angle *α* between the projection of the dipole onto the horizontal plane and a fixed axis in this plane (see Methods). On a qualitative level, the presence of a permanent dipole is immediately visible^39^: switching the field polarity makes the particle orientation flip (Video 2 in the Supplementary Material); no such flipping of particle orientation is possible for an isotropic sphere with an induced dipole^2^. To make a more systematic measurement, we follow the Brownian fluctuations of *α*, with the particle subject to a fixed *E*_0_. The experimental data (symbols in Fig. 4) clearly demonstrate that the standard deviation of *α* from its average value 
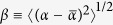
 is significantly reduced at large *E*_*ext*_. Clearly, for the particle Brownian rotation to be constrained by an external DC field, the particle must be carrying a permanent dipole. By thermodynamics, 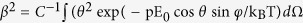, where 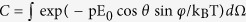, the integration is over the full 4*π* solid angle, and *θ* ∈ [−*π, π*]. Assuming that *pE*_0_ is simply proportional to *E*_*ext*_, we fit this expression to the experimental data, with only one free fitting parameter (*γ* ≡ *pE*_0_/*E*_*ext*_) involved. A very good agreement with the experiment is achieved (reduced *χ*^2^ ≈ 0.06), as demonstrated in Fig. 4 (solid curve). The fit quality significantly deteriorates, if pE_0_ is taken to be proportional to 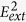, as for an induced^2^,^28^ dipole (dashes); the corresponding reduced *χ*^2^ value is 2.4-fold larger than for the permanent dipoles. Clearly, an induced dipole would not couple to spherical particle rotations, unless an easy polarization axis emerges due to structural defects or a possible (either chemical or physical) rotational anisotropy. The observed mismatch between the dashed curve and the experimental data rules out these more complex mechanisms, further supporting our conclusion that the particles bear permanent dipoles. In fluids of many particles, the presence of permanent dipoles makes the NN particles orient so as to minimize their dipole-dipole interaction energy. Oriented in such a way, the particles become attractive, giving rise to the potential well, shown in Fig. 2(b) and to the increased particle pair formation probability, demonstrated in the inset to Fig. 1(a).

While the chemical origin of the observed dipoles is yet unknown, they are likely to be caused by a slight variation of charge density across the surface of these colloids. Thus, the colloids cannot be described by point dipoles, such as in the Stockmayer fluid model^40^. The separation between the surfaces of adjacent particles, at relatively high *η* dealt with in the current work, is typically smaller than the particle size, so that the dipole approximation is invalid and multipolar contributions are significant. A full simulation of a fluid of such particles, with a microscopic model for the charge density variation on particle surfaces introduced, is beyond the scope of our current work. With that, remarkably, the increased probability of particle pair formation, observed in our experiments [inset to Fig. 1(a)], is readily reproduced by simple MD simulations of (WCA-repulsive) point-dipole-bearing spheres [inset to Fig. 2]. Unfortunately, with the expression for *β*^2^ (above) not allowing *E*_0_/*E*_*ext*_ and *p* to be decoupled, a direct quantitative comparison between the experimental and the theoretical magnitude of the dipoles cannot be at present carried out. However, these simulations of point-dipole-bearing spheres support our conclusion that surface charge nonuniformities at our particles’ surfaces may lead to effective attractions, calling for more advanced theoretical studies to be carried out in the future, allowing a full quantitative agreement with the experimental g(r) to be achieved.

## Conclusions

We have studied 2D fluids of PMMA colloidal spheres in an organic solvent, a simple model of the fluid Langmuir films. These studies, as also our direct measurements of the rotations of such particles in an external field, provide a strong evidence for such particles to have permanent electric dipoles. The pressure in the fluids of these particles, initially estimated by direct optical microscopy under an assumption of the interactions being hard, was compared to Volmer isotherms, virial expansions, and simulations, indicating significant deviations from the hard particle behavior. Furthermore, the microscopic structure and the range of bond-orientational order deviate from the behavior of hard or slightly-charged particles as well. We demonstrate that the observed deviations occur due to the dipole-dipole interactions between the colloids. These interactions are particularly important when the simple Coulomb repulsions between the particles are weak. With the chemical mechanism for dipole formation in organic solvents yet unknown, our work clearly calls for additional studies in this field to be carried out in the future. In particular, a wider range of different solvents should be used, with the dielectric constant varied from the currently-used *ε* ≈ 2 to that of water *ε* ≈ 80. We anticipate that such studies may possibly reveal unexpected links between the current work and the same-charge attractions in aqueous media^20^,^21^,^22^. Finally, our work opens new directions in self-assembly^17^,^28^ and may shed light onto the physical mechanisms behind the complex three-body forces^41^,^42^ and the unexpectedly fat-tailed cluster size distributions^26^ detected in some colloidal suspensions.

## Methods

### Colloidal fluids

To form two-dimensional colloidal fluids (left panel of Video 1 in the Supplementary Material), we suspend PMMA [poly-(methylmethacrylate)] spheres, fluorescently-labelled for confocal imaging by Nile Red dye, in dodecane (C_12_, Sigma-Aldrich, ≥99%). The particles are sterically stabilized by PHSA [poly-(hydroxystearic) acid], which minimizes the van der Waals attractions. Structures of three-dimensional fluid suspensions of such particles have been previously demonstrated to be perfectly described by the hard spheres’ model^43^, indicating that van der Waals attractions are negligible. The suspension is then loaded into a rectangular *Vitrocom* capillary (0.1 × 2 × 50 mm) and sealed with Epoxy glue. The shortest dimension of the capillary is oriented vertically, along the gravity, so that the particles sediment onto the bottom of the sample, forming a two-dimensional layer. The number density *ρ* of the particles in this layer is tunable, determined by the particles’ concentration in the initial suspension. To apply an external electric field, where relevant, electrodes are placed outside of the capillary, so as to have the field oriented along the shorter horizontal dimension of the capillary (2 mm). To completely avoid sticking of particles to the bottom of the capillary^26^, we add PHSA-co-PMMA comb stabilizer^44^ to the solvent at a concentration of 5% w/w. At smaller concentrations of PHSA-co-PMMA, its coverage of borosilicate capillary bottom by adsorption is insufficient, so that immobile colloids, permanently sticking to the glass, can be noticed. Free PHSA-co-PMMA, known to form micelles at higher concentrations in dodecane^45^, can potentially induce depletion attractions between the colloids. However, the range of such attractions is expected to be comparable to the radius of the micelles^7^,^45^, ~7 nm, far shorter than the length scales probed in our present work. In addition, experiments carried out with no free PHSA in the solution yield similar results; in these experiments, the capillary was first washed by a particle-free PHSA solution, allowing for the adsorption of PHSA to its walls, then dried, prior to loading of the PHSA-free sample. We determine the diameter of our particles by dynamic and static light scattering as *σ* = 2.4 *μm*; the size polydispersity of these particles^43^, measured by scanning electron microscopy, is ≤5%. The density mismatch between PMMA and C_12_ is ~0.3 g/cc, so that the particles are effectively confined to move in two dimensions, their gravitational length being smaller than *σ* by more than an order of magnitude^26^. We image these samples with a laser-scanning confocal fluorescence microscope (Nikon A1R) and 100 × 1.4NA oil immersion objective (Nikon) using either a galvanometric or a resonant scanning mode. We excite with *λ* = 514 nm and collect fluorescent images whose pixels correspond to 0.124 *μ*m in the sample. For particle location, we employ revised PLuTARC codes^19^, based on a modified version of the algorithm of Crocker and Grier, where tracking accuracy at high particle densities is significantly improved. To avoid uncontrolled sample drift under gravity during imaging^46^, the samples are mounted on a specially-designed tilt stage, where the angle between the short axis of the capillary and the gravity can be varied in the range between 0° and 2°, with the angular resolution being better than 0.1°. Samples tilted to a small angle with respect to gravity were left on the stage for equilibration. On equilibration, a small density gradient forms, akin to the profile in bulk suspensions at a sedimentation equilibrium, allowing the fluid at several different particle densities to be measured in a single preparation^4^. Several different samples were prepared and equilibrated at different angles, yielding identical fluid structures in all cases. The observed reproducibility strongly supports the validity of this experimental approach for fluids with short-range correlations, such as in the present work.

### Computer simulations

Event-driven Brownian dynamics (EDBD) simulations of HDs employed the open *HardBrown* codes^47^, compiled under Ubuntu. Typical system size included 1024 particles; simulations with larger and 4-fold smaller systems have been attempted, yielding identical results. At low concentrations, the initial configuration was random; at high concentrations, the simulation started from a square lattice configuration, complete relaxation of which was confirmed employing the visualization capabilities of *HardBrown*.

MD simulations employed LAMMPS^37^, running in the serial mode; a typical system included 40 × 40 particles. The simulations were carried out for two different types of pair potentials: (a) Weeks-Chandler-Andersen (WCA); (b) Full Lennard-Jones (LJ) potential. For WCA, which is a purely-repulsive truncated LJ,


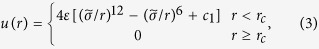


where *c*_1_ = 1/4 and 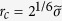. The parameters 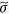 and *ε* are used to define convenient MD length and energy units, respectively, with the MD temperature *T* unit obtained by setting *k*_*B*_ = 1. The system was run at *T* = 1, for which the mean translational kinetic energy in 2D is unity. Periodic boundaries are used and the simulation cell size is determined by the overall number density. It should be pointed out that the MD approach is used here only to sample equilibrium configurations; the dynamics is not that of a colloidal system since the solvent is absent. Finally, the full LJ potential, including the attractions, was obtained from Eq. 3 by setting *c*_1_ = 0 and 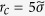. In simulations of dipole-bearing WCA spheres [inset to Fig. 2(b)], the dipole-dipole interaction was: 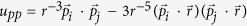, where 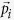 and 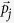 are the dipole moments of the *i*-th and the *j*-th particles, respectively. *u*_*pp*_ was cut off at *r* = 3*σ*.

## Additional Information

**How to cite this article**: Janai, E. *et al*. Dipolar colloids in apolar media: direct microscopy of two-dimensional suspensions. *Sci. Rep.* **6**, 28578; doi: 10.1038/srep28578 (2016).

## Supplementary Material

Supplementary Information

Supplementary Video 1

Supplementary Video 2
