# Supplementary material for: Dipolar colloids in apolar media: direct microscopy of two-dimensional suspensions
Source: Sci Rep. 2016 Jun 27;6:28578. doi: 10.1038/srep28578 (PMC4921927; doi:10.1038/srep28578)
Supplement: Supplementary Information [file srep28578-s1.pdf]

Dipolar colloids in apolar media: direct microscopy of two-dimensional suspensions

Erez Janai[1], Avner P. Cohen[1], Alexander V. Butenko[1], Andrew B. Schofield[2], Moty Schultz[1] and Eli Sloutskin[1,*]
[1]Physics Department and Institute for Nanotechnology & Advanced Materials, Bar-Ilan University, Ramat-Gan 5290002, Israel
[2]School of Physics and Astronomy, University of Edinburgh, Edinburgh EH9 3FD, United Kingdom}
[*]eli.sloutskin@biu.ac.il

Supplementary Information

Video 1. Left frame: raw confocal video of a colloidal 2D fluid, overlayed with red circles produced by the particle tracking software. Note the perfect agreement between the experimental image and the detected particle positions. Right frame: MD simulations of LJ particles. Note the different dynamics of the simulations, where ballistic, rather than Brownian motion occurs. Importantly, the span of data used for the actual *g(r)* analysis was much larger in time and spatial dimensions, for both experiments and computer simulations, allowing for very good statistics, such as in Fig. 1. The field of view is 62 × 62 μm. The raw data were collected at 4 fps.

Video 2. Raw confocal video of a particle in an external electric field. The fluorescent dye on one side of the particle is bleached, allowing the particle orientation (green arrow) to be manually tracked. The direction of the external field (blue arrow, in the top left corner) flips down towards the end of the movie. Note that the flipping of the external field makes the particle orientation immediately flip as well. No such flipping would have been observed for an isotropic sphere with an induced dipole. This fact provides a strong indication for our colloids to have permanent dipoles. The real time (in seconds) is shown at the bottom left corner of the image. The scale bar length (bottom right) is 1 μm.

<u>Inversion of the experimental *g(r)* to obtain *u(r)*</u>

To obtain *u(r)* from the experimental *g(r)*, we use the classical Ornstein-Zernike formalism with the hypernetted chain (HNC) closure approximation, following the procedure described in Ref. 20 in the main text. Note, Eq. (28) in Ref. 20 is fully correct, while a typo appears in this equation in some other papers by the same group (to the best of our knowledge, the calculations were, in all cases, carried out with the correct expression). In addition, to avoid spurious oscillations of *u(r)*, which may otherwise be occasionally observed even for *g(r)* of ideal hard disks, we start the iteration procedure with $I(r)=n^{-1}(u_{WCA}(r)+ln[g(r)])$, instead of $I(r)=0$ used in previous works; here u_{WCA} is the purely-repulsive and continuous WCA potential and $k_BT=1$.
